# Anomalous Origin of the Right Pulmonary Artery From the Ascending Aorta in a 10-Month-Old Child: A Case Report From Tanzania

**DOI:** 10.1177/2324709616648992

**Published:** 2016-05-09

**Authors:** Pedro Pallangyo, Frederick Lyimo, Paulina Nicholaus, Maria Mtolera

**Affiliations:** 1 The Jakaya Kikwete Cardiac Institute, Dar es Salaam, Tanzania; 2 Muhimbili National Hospital, Dar es Salaam, Tanzania

**Keywords:** anomalous pulmonary artery, hemitruncus arteriosus, congenital heart disease, pulmonary hypertension, congestive heart failure

## Abstract

Anomalous origin of the right pulmonary artery from the ascending aorta is a rare congenital deformity associated with poor quality of life and reduced life expectancy. Without a corrective surgery, less than one third of cases will live to see their sixth month. We report a case of a 10-month-old male child from Tanzania who presented with a 6-month history of recurrent respiratory tract infections, mild effort intolerance, and failure to thrive.

## Introduction

Anomalous origin of the right pulmonary artery from the ascending aorta (hemitruncus arteriosus) is among the very rare congenital anomalies constituting about 0.1% of all cardiac birth defects.^1,2^ Right pulmonary artery anomaly is 6 times more common than the left, and in about 60% of cases, hemitruncus occurs in isolation. Patent ductus arteriosus is the most frequent associated condition; however, other abnormalities, including tetralogy of Fallot, atrial septal defect, ventricular septal defect, and coarctation of aorta, have been documented.^2-4^

Although the pathophysiology of this anomaly is poorly understood, a partial or complete developmental failure of the left sixth arch is the established underlying etiology. Resulting from this, a large left-to-right shunt occurs with one lung receiving the entire cardiac output from the right ventricle while the other receives blood from the aorta at a systemic pressure, both resulting in pressure and/or volume overload.^4,5^ Hemitruncus arteriosus is frequently symptomatic from early infancy, usually presenting with recurrent respiratory tract infections, respiratory distress, congestive heart failure, pulmonary hypertension, and a failure to thrive.^2,3,5^ Surgical correction is the definitive treatment that ideally should be performed as urgently as possible following the diagnosis as mortality rates at 3 and 6 months are about 30% and 70%, respectively.^1,2,4^

## Case Report

A 10-month-old boy presented to our institution with a 6-month history of recurrent respiratory tract infections, mild effort intolerance, and failure to thrive. He was delivered vaginally at term in a twin pregnancy, weighed 3.0 kg, and had an APGAR score of 9/10. The second twin was also a male, weighed 2.5 kg, and had an APGAR score of 8/10. Both twins breastfed well, had an uneventful medical history for the first 3 months of life, and attained weights of 7.2 kg and 6.9 kg, respectively. From the fourth month onwards, the first twin started developing the aforementioned complaints, and the family sought medical attention from several health facilities. The child was diagnosed to have pneumonias and has been on several medications including amoxicillin, co-amoxiclav, and ceftriaxone with brief periods of symptoms cessation. With time, the child became progressively tachypneic and dyspneic but never cyanosed, had difficulties in breastfeeding, and started losing weight. The boy underwent 3 echocardiographic (ECHO) examinations in 3 different health facilities between the age of 8 and 10 months, all reporting normal findings.

Physical examination conducted at our institution revealed a small for age boy with some conjunctival pallor, but not jaundiced or cyanosed. The jugular venous pressure was not raised and there was no precordial hyperactivity. The blood pressure was 119/76 mm Hg, heart rate was 134 beats/min and regular, and apex beat was felt at the seventh intercostal space lateral to the midclavicular line. The first (S1) and second (S2) heart sounds were heard but auscultation of the pulmonic area revealed accentuated pulmonary component (P2); no murmur was heard. The respiratory rate was 38 breaths/min, oxygen saturation was 96% at room air, and auscultation of the chest revealed fine crepitations in the right lower zones. The child did not have finger clubbing, ascites, hepatomegaly, or lower limb edema.

**Figure 1.**
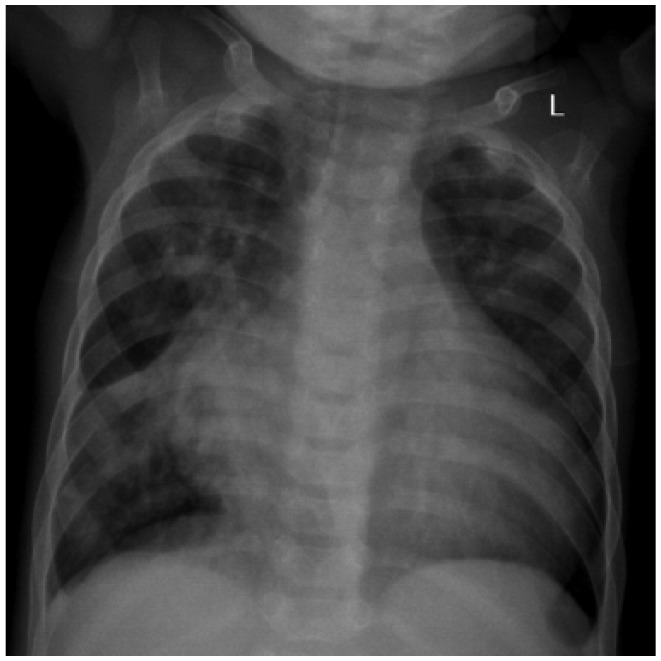
Chest X-ray (AP view) displaying cardiomegaly, increased vascular markings, and right lower lobe consolidation.

**Figure 2. fig2-2324709616648992:**
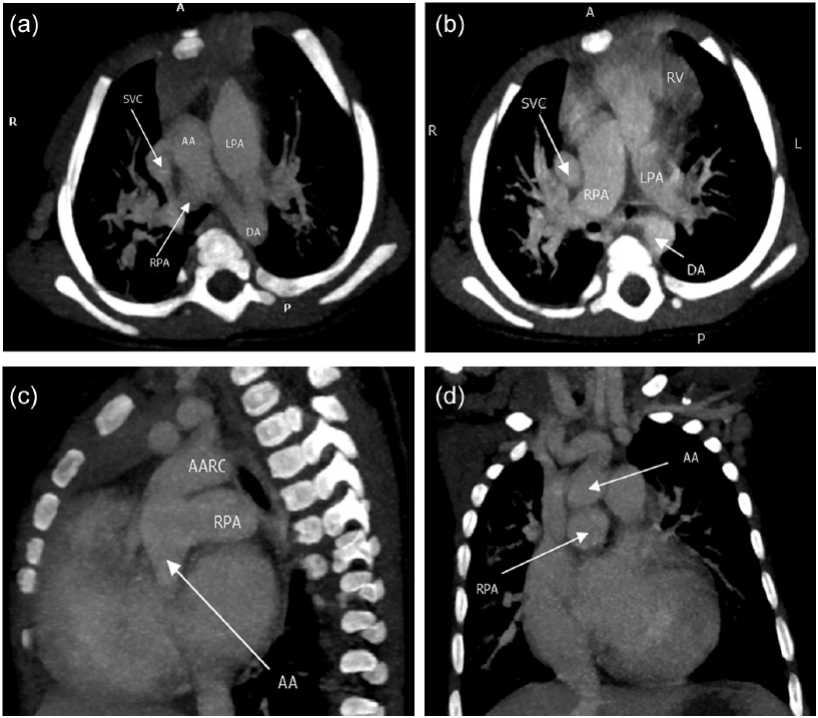
(a) Cardiac computed tomography scan (CT; axial view) showing right pulmonary artery arising from the ascending aorta. (b) Cardiac CT (axial view) showing a hemitruncus pulmonary artery. (c) Cardiac CT (sagittal view) showing right pulmonary artery arising from the ascending aorta. (d) Cardiac CT (coronal view) showing right pulmonary artery arising from the ascending aorta.

Hematological tests revealed iron deficiency anemia (hemoglobin 9.2 g/dL, mean corpuscular volume 54.8 fL, mean corpuscular hemoglobin 14.6 pg/cell, red cell distribution width 50.6 fL), leukocytosis (12.49 cells/µL) with lymphocyte predominance (66.9%) and thrombocytopenia (148 000/µL). Sickling test, RPR for syphilis, and ELISA for HIV were negative. Chest X-ray revealed cardiomegaly (cardiothoracic ratio 0.72), increased vascular markings in upper zones bilaterally, and right lower zone consolidation (Figure 1). An ECHO revealed a mild tricuspid regurgitation, mild pulmonary hypertension (35 mm Hg), biventricular hypertrophy, normal systolic function (ejection fraction 79%), and absence of a congenital anomaly. The child was further investigated with a cardiac computed tomography scan, which finally revealed the diagnosis of hemitruncus arteriosus (Figure 2a [Fig fig2-2324709616648992][Fig fig2-2324709616648992]-d).

The maximum weight attained by the first twin was 7.2 kg; however, currently the boy weighs 5.8 kg and can sit with support. In comparison, the second twin weighs 8.5 kg and can walk with support. Subsequently the boy was discharged home and continues to be attended as an outpatient on a weekly basis at the Jakaya Kikwete Cardiac Institute, while logistics for a referral overseas are ongoing. The child is relatively stable clinically and breastfeeds better than a couple of months ago; however, mild effort intolerance still persists. During his last visit, spironolactone, furosemide, hematenics, and amoxicillin syrup were prescribed.

## Discussion

Despite its rarity, early recognition of hemitruncus arteriosus is essential in providing optimal patient management owing to the excellent short- and long-term prognosis resulting from early corrective surgery.^6,7^ Several surgical techniques have been employed but the surgical division and direct anastomosis of the anomalously connected pulmonary artery is frequently reported.^6^ In contrast, palliative management, including pulmonary artery banding and aortopulmonary shunt, has been associated with a substantial increase in mortality.^6^

About two thirds of the reported corrective surgeries for hemitruncus were performed between the neonatal period and 6 months of age.^6^ This is so because within this age the progressive and irreversible pulmonary vascular disease is unlikely to have set in. Our patient started seeking medical attention from the age of 4 months, but disappointingly it took 6 months for a correct diagnosis to be reached. Intriguingly, our patient underwent 4 ECHO examinations and none of them seemed to have a suspicion of hemitruncus arteriosus. This case is a clear example of a missed diagnosis and inevitably a missed opportunity for an early surgical correction. Consequently, the prognosis in the case presented is poor as the already presence of pulmonary hypertension preclude a successful corrective surgery. Potentially, the child may benefit from a double lung transplant with the correction of right pulmonary artery origin. Apparently neither of the procedures can be performed at local institutions as of present and the procedure to secure a referral abroad through state sponsorship is lengthy.

As far as we can ascertain, this is the first case of anomalous origin of the right pulmonary artery from the ascending aorta to be reported in this set up. The authors hope that this case will provide a learning platform to practitioners and that subsequent cases of the like will be diagnosed promptly and managed in a timely and aggressive manner. In conclusion, despite its infrequency, infants with features suggestive of congenital heart disease should be assessed for hemitruncus arteriosus when more common conditions like patent ductus arteriosus, tetralogy of Fallot, atrial septal defect, and ventricular septal defect have been ruled out.
